# Western diet-induced hepatic steatosis and alterations in the liver transcriptome in adult Brown-Norway rats

**DOI:** 10.1186/s12876-015-0382-3

**Published:** 2015-10-30

**Authors:** Michael D. Roberts, C. Brooks Mobley, Ryan G. Toedebush, Alexander J. Heese, Conan Zhu, Anna E. Krieger, Clayton L. Cruthirds, Christopher M. Lockwood, John C. Hofheins, Charles E. Wiedmeyer, Heather J. Leidy, Frank W. Booth, R. Scott Rector

**Affiliations:** School of Kinesiology, Auburn University, Auburn, AL USA; Edward Via College of Osteopathic Medicine-Auburn Campus, Auburn, AL USA; Department of Biomedical Sciences, University of Missouri, Columbia, MO USA; 4Life Research, Sandy, UT USA; Department of Veterinary Pathobiology, University of Missouri, Columbia, MO USA; Department of Nutrition and Exercise Physiology, University of Missouri, Columbia, MO 65212 USA; Department of Medical Pharmacology and Physiology, University of Missouri, Columbia, MO USA; Dalton Cardiovascular Research Center, University of Missouri, Columbia, MO USA; Department of Medicine-Gastroenterology and Hepatology, University of Missouri, Columbia, MO USA; Research Service-Harry S Truman Memorial VA Hospital, Columbia, MO USA

**Keywords:** High-fat diet, Sucrose, Fatty liver, Inflammation, RNA-seq

## Abstract

**Background:**

The purpose of this study was to investigate the effects of sub-chronic high fat, high sucrose diet (also termed ‘Westernized diet’ or WD) feeding on the liver transcriptome during early nonalcoholic fatty liver disease (NAFLD) development.

**Methods:**

Brown Norway male rats (9 months of age) were randomly assigned to receive ad libitum access to a control (CTL; 14 % kcal fat, 1.2 % sucrose by weight) diet or WD (42 % kcal from fat, 34 % sucrose by weight) for 6 weeks.

**Results:**

Six weeks of WD feeding caused hepatic steatosis development as evidenced by the 2.25-fold increase in liver triacylglycerol content, but did not induce advanced liver disease (i.e., no overt inflammation or fibrosis) in adult Brown Norway rats. RNA deep sequencing (RNA-seq) revealed that 94 transcripts were altered in liver by WD feeding (46 up-, 48 down-regulated, FDR < 0.05). Specifically, the top differentially regulated gene network by WD feeding was ‘Lipid metabolism, small molecular biochemistry, vitamin and mineral metabolism’ (Ingenuity Pathway Analysis (IPA) score 61). The top-regulated canonical signaling pathway in WD-fed rats was the ‘Superpathway of cholesterol biosynthesis’ (10/29 genes regulated, *p* = 1.68E-17), which coincides with a tendency for serum cholesterol levels to increase in WD-fed rats (*p* = 0.09). Remarkably, liver stearoyl-CoA desaturase (*Scd*) mRNA expression was by far the most highly-induced transcript in WD-fed rats (approximately 30-fold, FDR = 0.01) which supports previous literature underscoring this gene as a crucial target during NAFLD development.

**Conclusions:**

In summary, sub-chronic WD feeding appears to increase hepatic steatosis development over a 6-week period but only induces select inflammation-related liver transcripts, mostly acute phase response genes. These findings continue to outline the early stages of NAFLD development prior to overt liver inflammation and advanced liver disease.

## Background

Many modernized cultures consume a high-fat, high sugar diet rich in calories, termed a Western diet (WD), which contributes to obesity-related maladies. Concomitant with the staggering rise in the obesity epidemic is the prevalence of nonalcoholic fatty liver disease (NAFLD) which is now estimated to affect ~30 % of the adult population in the United States, and is present in 75–100 % of obese or morbidly obese individuals [1, 2]. NAFLD is a progressive liver disease ranging from simple steatosis, nonalcoholic steatohepatitis (NASH), fibrosis, and cirrhosis [3]. It is characterized by elevated hepatic triglyceride (TG) storage (≥5 % by weight) in the absence of excessive alcohol consumption (<20 g/d) and is considered to be the hepatic manifestation of the metabolic syndrome (reviewed in [3]).

Western Diet (WD) feeding in rodents leads to the rapid development of NAFLD and subsequent development of NASH [4]. High-fat and/or high-sugar feeding studies examining the liver transcriptome have reported that inflammatory and lipid metabolism markers increase while oxidative stress defense markers decrease following 4–20 weeks of high fat diet feeding [5–8]. A more recent publication examining numerous transcriptomic studies identified 31 hepatic gene candidates that are induced in mice following a high fat diet [9]; specifically, mRNAs related to fatty acid β-oxidation, fatty acid synthesis, and gluconeogenesis were up-regulated; whereas, genes involved in sterol biosynthesis, insulin signaling, and oxidative stress defense were down-regulated following high fat feeding. Therefore, it is apparent that high-fat/high-sugar diets exert profound and rapid alterations in the liver transcriptome which precede and/or coincide with disease progression.

We recently performed a sub-chronic (6-week) feeding study examining the effects of an antioxidant supplement on markers of liver health during concomitant WD feeding in 9-month old adult Brown-Norway rats [10]. When comparing WD *ad libitum*-fed rats versus rats provided control diet (CTL; low fat, low sugar) groups, we found pronounced hepatic lipid accumulation, as determined by Oil Red O and H&E staining, suggestive of a NAFLD phenotype. However, it remains to be determined if lipid accumulation with sub-chronic WD feeding altered or coincided with serum and liver markers suggestive of a pro-inflammatory response. Furthermore, there are a limited number of investigations having examined global transcriptomic changes in the liver during the early stages of WD feeding. The information will lead to better understanding of early progression of fatty liver disease, and will help to identify potential interventional targets. Therefore, the purpose of the current study was to examine how 6 weeks of WD versus CTL feedings in adult Brown Norway rats: a) affects the liver transcriptome, and b) affects select serum and liver pro-inflammatory and fibrotic markers.

## Methods

### Animals and dietary feeding paradigm

All experimental protocols were approved by the University of Missouri’s Animal Care and Use Committee. Nine-month-old male Brown Norway rats (Charles River Laboratory, O’ Fallon, Missouri) were assigned to one of the following two groups for 6 weeks: 1) *ad libitum* WD feeding (WD; *n* = 8); and 2) *ad libitum* low-fat/low sugar diet (CTL; *n* = 6). WD (TD.88137; Harlan Laboratories) contained the following nutrient make-up: 4.5 kcal/g, 15.2 % protein (expressed as %kcal), 42.7 % carbohydrate (expressed as %kcal), 42.0 % fat (expressed as %kcal), and 0.2 % cholesterol (by weight). Of note, this diet has been used in prior rodent studies to elicit NASH [11, 12]. CTL (LabDiet_®_ Certified CR 14 % protein rodent diet) contained the following nutrient make-up: 3.5 kcal/g, 16.1 % protein (expressed as %kcal), 69.3 % carbohydrate (expressed as %kcal), 14.6 % fat (expressed as %kcal), and 0.014 % cholesterol (by weight). WD also contained a high proportion of sucrose (34 % of the total diet by weight), whereas CTL did not (1.2 % of the total diet by weight).

Rats were singly housed in standard rat cages in temperature-controlled animal quarters (21 °C) with a 0700–1900 light: 1900–0700 dark cycle that was maintained throughout the experimental period. Food intakes were carefully measured weekly (i.e., monitoring cage bottoms for morsels, etc.) and total caloric intakes for the duration of the study have been reported elsewhere [10].

### Euthanasia procedures

Euthanasia procedures have been described in detail elsewhere [10]. Briefly, on the day of euthanasia, animal cages were removed from the animal quarters between 0800–0900 and food was removed from each cage. Euthanasia took place between 1400–1900 and rats were sacrificed under CO_2_ gas in their home cages in order to minimize stress. Whole blood was subsequently removed via heart sticks using a 21-gauge needle and syringe, placed in a serum separator tube, centrifuged at 3200 rev/min for 5 min, serum was aliquoted into 1.7 ml microcentrifuge tubes, and serum was flash frozen for later serum cytokine assessment. Separate median lobe liver sections (~50–100 mg) were subsequently removed using standard dissection techniques and were flash frozen in liquid nitrogen for RNA deep-sequencing procedures, NF-κB pathway phosphoprotein analysis, and liver trichrome staining and triacylglycerol (TAG) analysis respectively.

### Liver trichrome staining and TAG content

Histochemical intrahepatic TAG content was determined as previously described [13]. To examine liver morphology, formalin-fixed, paraffin-embedded livers were sectioned and stained with trichrome stain for collagen deposition/fibrosis.

### Liver RNA-seq methods

#### RNA isolation and cDNA preparation

Two micrograms of liver RNA from WD (*n* = 6) and CTL (*n* = 6) rats were sent to the University of Missouri’s DNA Core for RNA-seq procedures. Of note, only 6 of the 8 WD rats were used for RNA-seq due to resource constraints. High RNA integrity of each sample was confirmed using the BioAnalyzer 2100 automated electrophoresis system (Bio-Rad, Hercules, CA, USA) prior to cDNA library construction. cDNA library preparation was subsequently performed using the manufacturer’s protocol with reagents supplied in Illumina’s TruSeq RNA sample preparation kit v2. Poly-A containing mRNA was purified from 2 μg of total RNA, RNA was fragmented, double-stranded cDNA was generated from fragmented RNA and the index containing sample identifier adapters were ligated to the ends. The final construct of each purified library was evaluated using the BioAnalyzer 2100 automated electrophoresis system, quantified with the Qubit fluorometer using the quant-iT HS dsDNA reagent kit (Invitrogen, Life Technologies, Grand Island, NY), and diluted according to Illumina’s standard sequencing protocol for sequencing on the HiSeq 2000.

#### Illumina sequencing of NAc cDNA and statistical analyses of RNA-seq data

RNA-seq procedures occurred at the University of Missouri DNA Core and are described in more detail elsewhere [14]. Briefly, following cDNA library construction, samples were loaded onto a flow cell where clusters of each oligo were replicated. Following this procedure, flow cells were placed in the sequencer and fluorescently-labeled bases were attached to the complementary bases of each sequence. The Illumina Genome Analyzer recorded 50 bp reads. Reads were trimmed to ensure adaptor sequence removal and tiled to a custom reference using NextGENe v1.92 (SoftGenetics, State College, PA).

### Liver NF-κB pathway phosphoprotein determination

Briefly, 50–100 mg of excised liver tissue was homogenized on ice in RIPA buffer [50 mM Tris–HCl (pH 8.0), 150 mM NaCl, 1 % NP-40, 0.5 % sodium deoxycholate, 1 % SDS, 1x protease inhibitor, phosphatase II and III inhibitor cocktails (Sigma, St. Louis, MO, USA)] using a Tissuelyser (Qiagen, Valencia, CA, USA) at 20 Hz for 1 min. The homogenate was centrifuged at 12,000 g for 10 min and the resultant supernatant was obtained for Western blotting. Protein concentrations were obtained using the BCA assay (Pierce Biotechnology, Rockford, IL) and 60 μg of protein in loading buffer was loaded onto 18 % SDS-PAGE gels. Proteins were transferred onto PVDF membranes and primary antibodies [rabbit monoclonal phospho-IkBα (Ser32) at 1:1,000, pan mouse monoclonal IkBα at 1:1,000, pan rabbit monoclonal phospho-NF-κB (Ser536) at 1:1,000, and pan rabbit monoclonal phospho-NF-κB at 1:1,000 (Cell Signaling, Danvers, MA, USA)], diluted in Tris-buffered saline + Tween20 with 5 % bovine serum albumin were applied to membranes overnight at 4 °C. HRP-conjugated secondary antibody (1:2,000; Cell Signaling), were applied for 1 h at room temperature, and ECL substrate (Pierce Biotechnology) was then applied for 5 min prior to exposure. Band densitometry was performed through the use of Kodak 4000R Imager and Molecular Imagery Software (Kodak Molecular Imaging Systems, New Haven, CT).

### Serum cytokine profile determination

Serum samples were assayed in duplicate for concentrations of leptin, IL-1β, IL-6, MCP-1, and TNF-α using a multiplex cytokine assay (Millipore Milliplex, cat no. RCYTOMAG-80 K; Billerica, MA, USA) on a MAGPIX instrument (Luminex Technologies; Luminex, Austin, TX, USA) according to the manufacturer’s instructions.

### Statistics and bioinformatics for liver RNA-seq data

Differential gene expression patterns were analyzed for annotated genes between the WD and CTL groups using reads per kilobase per million mapped reads (RPKM) values. Differentially expressed liver mRNAs were considered to be significant when WD/CTL fold-change false discovery rate (FDR) p-values were < 0.05. Liver transcripts that were found to be altered with WD feeding were subsequently entered into Ingenuity Pathway Analysis (Qiagen) to elucidate gene networks and/or biological functions within the liver that were altered.

### Statistics for non-RNA-seq data

Unless otherwise stated, dependent variables are presented as mean ± standard error and between-group comparisons were performed using independent samples t-tests. Significance was set at an alpha level of 0.05.

## Results

### Phenotypic changes following 6 weeks of WD versus CTL feeding

Phenotypic changes in WD versus CTL rats are presented in Table 1 below. As reported previously [10], WD feeding significantly increased body mass at sacrifice by 6 % (*p* = 0.0019), omental fat pad mass by 52 % (*p* < 0.001), perirenal fat pad mass by 74 % (*p* < 0.001), and liver fat deposition by 4.2-fold (*p* = 0.004). We also previously reported that WD rats consumed 15 % more calories compared to CTL rats (*p* < 0.001), with a diet higher in total fat, total sucrose, and total cholesterol. We also expand upon our previous findings of increases in neutral lipid staining by Oil-Red O in WD rats by reporting that liver TAG content was 2.2 fold higher in WD versus CTL rats (*p* < 0.001; Table 1). Moreover, trichrome staining revealed no difference in liver fibrosis in WD versus CTL rats (data not shown).

**Table 1:** Animal characteristics following 6 weeks of WD versus CTL feeding

Variable	CTL	WD	*p*-value
*Body and tissue masses*			
Body mass at sacrifice (g)	329 ± 7	350 ± 12	0.002
Omental fat mass (g)	0.25 ± 0.03	0.38 ± 0.03	<0.001
Perirenal fat mass (g)	1.70 ± 0.14	2.95 ± 0.40	<0.001
*Dietary data*			
Total kcal consumed	2,488 ± 32	2,849 ± 38	<0.001
Total protein consumed (g)	100 ± 1	110 ± 1	<0.001
Total carbohydrate consumed (g)	461 ± 6	307 ± 4	<0.001
Total fat consumed (g)	40 ± 1	134 ± 2	<0.001
Total sucrose consumed (g)	8.5 ± 0.1	215 ± 3	<0.001
Total cholesterol consumed (g)	0.099 ± 0.001	1.27 ± 0.02	<0.001
Liver characteristics			
Fat deposition (Oil-Red O area; AUs)	167.6 ± 51.3	701.3 ± 139.5	0.004
Liver TAGs (nmol/g tissue)	3.34 ± 0.25	7.53 ± 0.58	<0.001

Values are means ± SE. Body mass, total kcal consumed, and Oil-Red O data previously reported [10]; symbols: g, grams; AUs, arbitrary units

### WD feeding disrupts the hepatic expression of genes involved with cholesterol and fatty acid synthesis

**Fig. 1:**
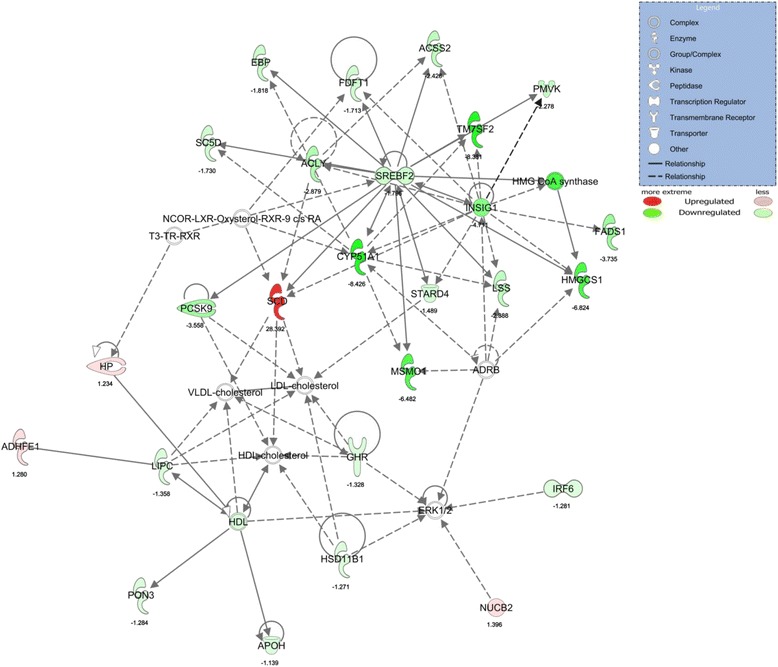
The top regulated gene network in WD versus CTL rats. The top regulated gene network in WD versus CTL rats included ‘Lipid metabolism, small molecular biochemistry, vitamin and mineral metabolism’ (IPA score 61; Fig. 1). Specific hubs down-regulated (green) in WD were *Srebp2*, *Insig1*, and *Cyp51a1. Scd* is the only molecule in the shown network to be up-regulated. Molecules shown in the network are: *Acly*, ATP citrate lyase; *Acss2*, acyl-CoA synthetase short-chain family member 2; *Adhfe1*, alcohol dehydrogenase, iron containing, 1; *Apoh*, apolipoprotein H (beta-2-glycoprotein I); *Cyp51a1*, cytochrome P450, family 51, subfamily A, polypeptide 1; *Ebp*, emopamil binding protein (sterol isomerase); *Fads1*, fatty acid desaturase 1; *Fdft1*, farnesyl-diphosphate farnesyltransferase 1; *Ghr*, growth hormone receptor; *Hmgcs1*, 3-hydroxy-3-methylglutaryl-CoA synthase 1 (soluble); *Hp*, haptoglobin; *Hsd11b1*, hydroxysteroid (11-beta) dehydrogenase 1; *Insig1*, insulin induced gene 1; *Irf6*, interferon regulatory factor 6; *Lipc*, hepatic lipase, hepatic; *Lss*, lanosterol synthase (2,3-oxidosqualene-lanosterol cyclase); *Msmo1*, methylsterol monooxygenase 1; *Nucb2*, nucleobindin 2; *Pcsk9*, proprotein convertase subtilisin/kexin type 9; *Pmvk*, phosphomevalonate kinase; *Pon3*, paraoxonase 3; *Sc5d*, sterol-C5-desaturase; *Scd*, stearoyl-CoA desaturase (delta-9-desaturase); *Srebf2*, sterol regulatory element binding transcription factor 2; *Stard4*, StAR-related lipid transfer (START) domain containing 4; *Tm7sf2*, transmembrane 7 superfamily member 2

**Fig. 2:**
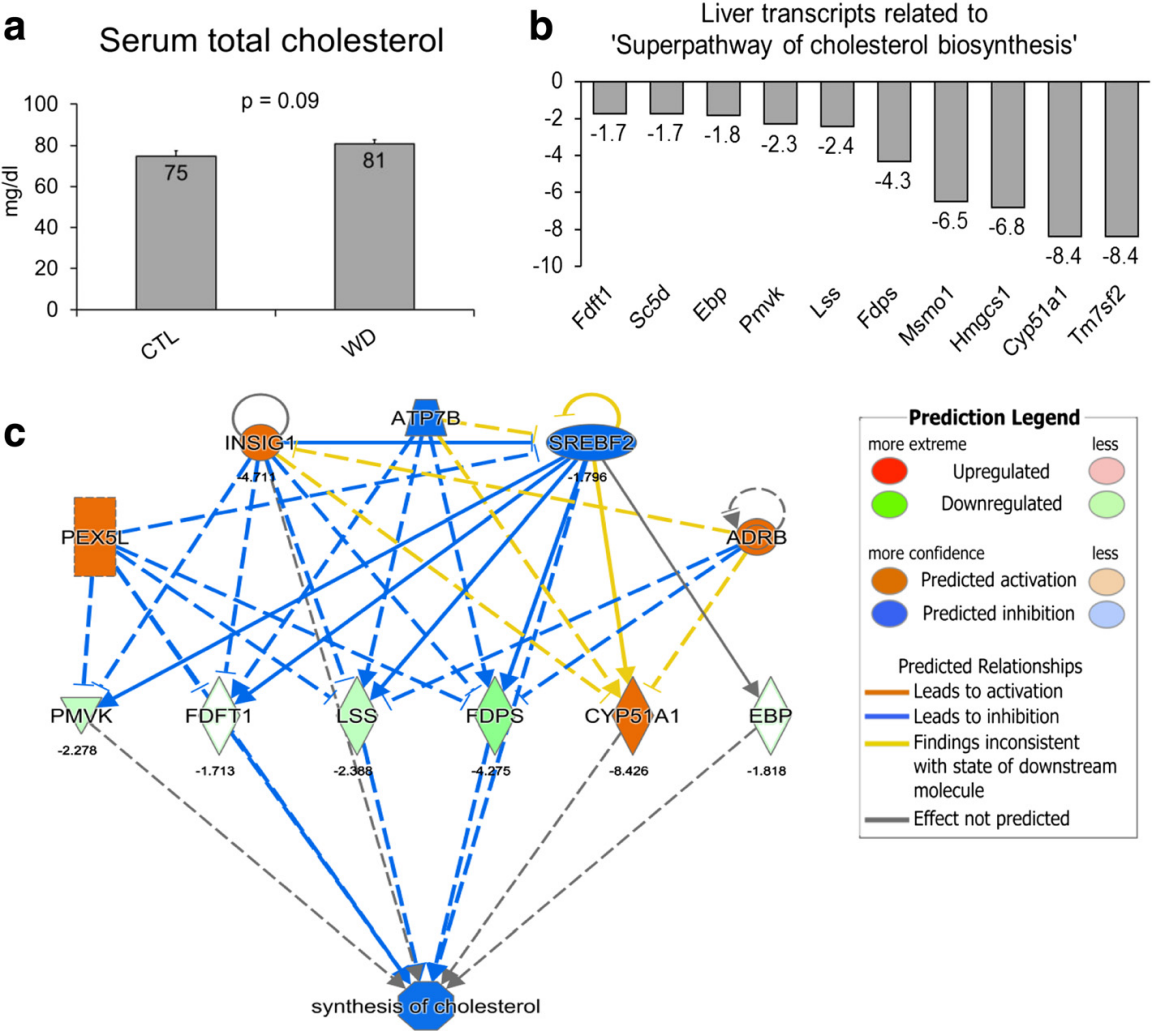
WD feeding dysregulates hepatic cholesterol biosynthesis gene expression. Values are means ± SE for Panel **a**. Total serum cholesterol (**a**) tended to be higher in WD. and transcript levels of a number of cholesterol biosynthesis genes unaltered with WD feeding (**b**, all responses *p <* 0.05). Top Regulator Effector Network in IPA (**c**) involved in the regulation of cholesterol biosynthesis. *Cyp51a1*, cytochrome P450, family 51, subfamily A, polypeptide 1; *Ebp*, emopamil binding protein (sterol isomerase); *Fdft1*, farnesyl-diphosphate farnesyltransferase 1; *Fdps*, farnesyl diphosphate synthase; *Hmgcs1*, 3-hydroxy-3-methylglutaryl-CoA synthase 1; *Lss*, lanosterol synthase (2,3-oxidosqualene-lanosterol cyclase); *Msmo1*, methylsterol monooxygenase 1; *Pmvk*, phosphomevalonate kinase; *Sc5d*, sterol-C5-desaturase; *Tm7sf2*, transmembrane 7 superfamily member 2; *Adrb*, Beta Adrenergic Receptor; *Atp7b*, ATPase, Cu++ transporting, beta polypeptide; *Insig1*, insulin induced gene 1; *Pex5l*, peroxisomal biogenesis factor 5-like; *Srebf2*, sterol regulatory element binding factor 2

Using a statistical cut-off of FDR < 0.05, we demonstrate that 6 weeks of WD feeding up-regulated 46 liver transcripts and down-regulated 48 transcripts. IPA readouts demonstrate that the top regulated gene network in WD versus CTL rats included ‘Lipid metabolism, small molecular biochemistry, vitamin and mineral metabolism’ (IPA score 61; Fig. 1). Serum cholesterol levels tended to be higher in WD-fed rats as previously reported [10] (Fig. 2a), which coincides with the aforementioned RNA-seq readout suggesting a dysregulation in cholesterol biosynthesis-related transcripts in WD versus CTL rats (Fig. 2b). Interestingly, the top regulated canonical signaling pathway in WD versus CTL rats was the ‘Superpathway of cholesterol biosynthesis’ (10/29 genes down-regulated, *p* = 1.68E-17; Fig. 2c).

Of the transcripts meeting the statistical cut-off of FDR < 0.05, only 4 mRNAs were up-regulated > 2.0-fold between WD versus CTL rats, whereas 17 mRNAs were down-regulated > −2.0-fold (Table 2); most of these being involved with cholesterol biosynthesis (WD/CTL mRNA fold change: *Cyp51a1* −8.43, *Tm7sf2* −8.39, *Hmgcs1* −6.82, *Msmo1* −6.48, *Insig1* −4.71, *Fdps −*3.74, *Pcsk9* −3.56, *Lss −*2.39, *Pmvk −*2.28), fatty acid synthesis (WD/CTL mRNA fold change: *Scd* +28.4, *Fads1* −3.74, *Acly −*2.88, *Acss2* −2.43) and drug metabolism (WD/CTL mRNA fold change: *Cyp2c18* + 2.50, *Cyp51a1* −8.43, *Ugt2a3* −2.13).

**Table 2:** Up- and down-regulated annotated liver transcripts on a fold-change basis with WD feeding

Transcript	Protein function	WD/CTL fold-change	FDR value	Nominal *p*-value
*Top transcripts up-regulated with WD feeding (FDR < 0.05, > 2.0 fold-change)*				
Stearoyl-CoA desaturase (delta-9-desaturase) (*Scd*)	Involved in fatty acid biosynthesis, primarily the synthesis of oleic acid; linked to inhibition of CPT-1A and suppression of mitochondrial fatty acid oxidation	28.392	0.01	0.00004
PDZ and LIM domain 1 (*Pdlim1*)	Cytoskeletal protein that may act as an adapter that brings other proteins (like kinases) to the cytoskeleton	2.741	0.006	0.00002
Cytochrome P450, family 2, subfamily C, polypeptide 18 (*Cyp2c18*)	This gene encodes a member of the cytochrome P450 superfamily of enzymes which are monooxygenases that catalyze reactions involved in drug metabolism and synthesis of cholesterol, steroids and other lipids	2.504	0.01	0.00003
Platelet factor 4 (*Pf4*)	Chemotactic molecule for neutrophil and monocyte attraction	2.031	0.04	0.0004
*Top transcripts down-regulated with WD feeding (FDR < 0.05, < −2.0 fold-change)*				
Cytochrome P450, family 51, subfamily A, polypeptide 1 (*Cyp51a1*)	This gene encodes a member of the cytochrome P450 superfamily of enzymes which are monooxygenases that catalyze reactions involved in drug metabolism and synthesis of cholesterol, steroids and other lipids	−8.426	0.0001	0.00000006
Transmembrane 7 superfamily member 2 (*Tm7sf2*)	Involved in the conversion of lanosterol to cholesterol	−8.381	0.007	0.00002
3-hydroxy-3-methylglutaryl-CoA synthase 1 (*Hmgcs1*)	This enzyme condenses acetyl-CoA with acetoacetyl-CoA to form HMG-CoA, which is the substrate for HMG-CoA reductase	−6.824	0.005	0.00001
Methylsterol monooxygenase 1 (*Msmo1*)	The protein is localized to the endoplasmic reticulum membrane and is believed to function in cholesterol biosynthesis	−6.482	0.002	0.000002
Zinc finger protein 48 (*Znf48*)	May be involved in transcriptional regulation	−6.458	0.00006	0.00000001
Insulin induced gene 1 (*Insig1*)	Mediates feedback control of cholesterol synthesis	−4.711	0.03	0.0002
RUN and SH3 domain containing 1 (*Rusc1*)	Seems to be involved in signaling pathways that are regulated by the prolonged activation of MAPK; may be involved in regulation of the NF-kappa-B pathway	−4.705	0.0001	0.00000008
Farnesyl diphosphate synthase (*Fdps*)	Involved in cholesterol synthesis	−4.275	0.0003	0.0000003
Fatty acid desaturase 1 (*Fads1*)	Component of a lipid metabolic pathway that catalyzes biosynthesis of unsaturated fatty acids polyunsaturated fatty acids	−3.735	0.004	0.000007
Proprotein convertase subtilisin/kexin type 9 (*Pcsk9*)	Acts via a non-proteolytic mechanism to enhance the degradation of the hepatic low-density lipoprotein receptor	−3.558	0.0001	0.0003
Cysteine sulfinic acid decarboxylase (*Csad*)	Plays a role in multiple biological processes as the rate-limiting enzyme in taurine biosynthesis	−3.023	0.049	0.0005
ATP citrate lyase (*Acly*)	ATP citrate-lyase is the primary enzyme responsible for the synthesis of cytosolic acetyl-CoA in many tissues; has a central role in de novo lipid synthesis	−2.879	0.049	0.0006
Acyl-CoA synthetase short-chain family member 2 (*Acss2*)	Activates acetate so that it can be used for lipid synthesis or for energy generation	−2.426	0.0001	0.00000003
Lanosterol synthase (2,3-oxidosqualene-lanosterol cyclase) (*Lss*)	Catalyzes the cyclization of (S)-2,3 oxidosqualene to lanosterol, a reaction that forms the sterol nucleus	−2.388	0.02	0.00008
Deoxyribonuclease II beta (*Dnase2b*)	Gene is ubiquitously expressed but liver function unknown	−2.344	0.02	0.0002
Phosphomevalonate kinase (*Pmvk*)	Involved in the cholesterol biosynthetic pathway	−2.278	0.004	0.000006
UDP glucuronosyltransferase 2 family, polypeptide A3 (*Ugt2a3*)	UDP-glucuronosyltransferases are involved in the elimination of potentially toxic xenobiotics and endogenous compounds	−2.129	0.009	0.00003

### WD feeding does not affect systemic or hepatic pro-inflammatory markers, but modestly affects markers of the acute phase protein response

**Fig. 3:**
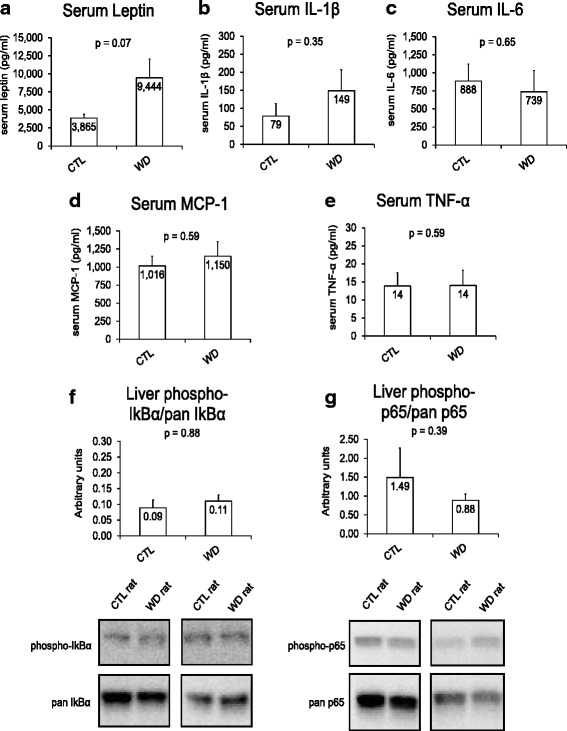
Effects of WD feeding on systemic and hepatic pro-inflammatory markers. Serum concentrations of leptin (**a**), IL-1β (**b**), IL-6 (**c**), MCP-1 (**d**), and TNF-α (**e**). Hepatic protein content for phospho-IkBα and phospho-p65 (**f** & **g**). Values are means ± SE. WD feeding tended to elevate serum leptin compared to CTL rats (*p* = 0.07), while not affecting other pro-inflammatory serum markers. Moreover, WD feeding did not affect liver markers of NF-κB signaling

Surprisingly, six weeks of WD feeding did not significantly affect serum cytokines (Fig. 3a-e) and/or liver phospho-IkBα and/or phospho-NF-κB p65 patterns (Fig. 3f/g). Likewise, RNA-seq readouts for genes related to TNF-α signaling, IL-6 signaling, and IL-1β signaling suggest that 6 weeks of WD feeding only elicits modest alterations in pro-inflammatory cytokine pathway signaling genes (Table 3). Specifically, *IL-1β* mRNA and *Jak3* mRNA (IL-6 downstream effector) were up-regulated in WD versus CTL rats (nominal p-value < 0.05), albeit these increases did not meet the FDR cut-off values. Interestingly, WD feeding caused a differential regulation of liver mRNAs related to the acute phase response (Table 3). Specifically, WD feeding caused an up-regulation in platelet factor 4 (*Pf4*) mRNA (Table 3) which is a putative chemoattractant protein that recruits neutrophils and monocytes. When examining a spectrum of acute phase response genes, WD feeding also significantly increased serum amyloid P component (*Apcs*) mRNA, haptoglobin (*Hb*) mRNA and superoxide dismutase 2 (*Sod2*) mRNA while significantly decreasing apolipoprotein H (*Apoh*) mRNA and lipopolysaccharide binding protein (*Lpb*) mRNA. Finally, WD feeding down-regulated the mRNA expression of the growth hormone receptor (*Ghr*) (WD/CTL: −1.33, FDR = 0.04). This is of particular interest given that: a) the liver is a very responsive tissue to growth hormone, and b) the macrophage-specific knockout of *Ghr* causes an enhanced inflammatory response in peripheral tissues when animals were challenged with a high-fat diet [15]. Collectively, these modest transcript alterations may be initial changes that occur with the development of WD-induced liver inflammation that has been shown to occur with longer feeding schedules [16, 17].

**Table 3:** Select liver transcripts in WD versus CTL rats related to pro-inflammatory cytokine, acute-phase protein response, and macrophage infiltration

Transcript	CTL RPKM mean	WD RPKM mean	WD/CTL Fold-change	Nominal *p*-value
*Select genes involved in IL-6 signaling*				
*IL-6*	not on RNA-seq readout			
*IL-6R*	29.55	30.89	1.05	0.80
*gp130*	26.04	23.44	−1.11	0.44
*Jak1*	34.9	34.1	−1.02	0.66
*Jak2*	1.87	1.81	−1.03	0.78
***Jak3***	**2.02**	**2.78**	**1.37**	**0.034**
*Stat3*	15.43	16.20	1.05	0.68
*Socs3*	2.49	3.44	1.38	0.47
*Select genes involved in TNF-α signaling*				
*TNF-α*	0.02	0.03	1.56	0.38
*Tnfrsf1b*	3.91	2.88	−1.36	0.08
*Traf2*	6.96	7.06	1.01	0.81
*Rela (p65)*	10.56	10.64	1.01	0.89
*Nfkbia (IkBa)*	18.57	18.35	−1.01	0.90
*Select genes involved in IL-1β signaling*				
***IL-1β***	**1.99**	**2.93**	**1.47**	**0.013**
*IL-1R1*	6.38	6.79	1.07	0.75
*Traf6*	1.45	1.30	−1.12	0.23
*Mapk8 (JNK1)*	2.24	1.98	−1.13	0.32
*Mapk9 (JNK2)*	18.27	19.25	1.05	0.52
*Select genes encoding acute phase response proteins*				
***Apcs***	**1048**	**862**	**−1.22**	**0.047**
***Apoh***	**3874**	**3401**	**−1.07**	**0.006**
*C2*	51.0	47.0	−1.09	0.10
*C9*	517	614	1.19	0.10
*Crp*	not on RNA-seq readout			
*F2*	1051	1060	1.01	0.86
*Fth1*	1817	1969	1.08	0.18
*Hmox1*	16.3	17.0	1.05	0.62
***Hp***	**1299**	**1603**	**1.24**	**0.0002**
*Hpx*	2667	3086	1.16	0.11
*IL-1rn*	13.7	11.5	−1.19	0.35
***Lbp***	**538**	**350**	**−1.54**	**0.002**
*Mur2*	432	392	−1.10	0.31
***Pf4***	**2.48**	**5.04**	**2.03**	**0.0004**
*Plg*	944	868	−1.08	0.11
***Sod2***	**41.8**	**47.2**	**1.13**	**0.013**
*Select genes representative of macrophage markers*				
*Cd68*	12.69	12.94	1.02	0.86
*Emr1 (F4/80)*	4.33	4.89	1.13	0.32
***Itgax (Cd11c)***	**0.27**	**0.42**	**1.53**	**0.044**
*Itgam (Cd11b)*	0.37	0.37	−1.01	0.92

Bold-faced transcript indicates that nominal *p*-values < 0.05

## Discussion

These RNA-seq data-mining efforts provide a unique snapshot of liver transcriptomic alterations that occur with sub-chronic WD feeding in adult Brown Norway rats. While it has demonstrated that longer high-fat/high-sugar feeding schedules cause increases in liver inflammation [17], oxidative stress [18], fibrosis [17, 18] and mitochondrial dysfunction [19, 20], we here provide evidence that sub-chronic WD feeding causes a rapid dysregulation in select hepatic lipid and cholesterol metabolism genes in rats; an effect which: a) may contribute to the doubling in hepatic TG content presented herein; and b) may be responsible for serum cholesterol levels tending to increase after 6 weeks of WD feeding as previously reported [10]. Furthermore, the RNA-seq analyses reveal that while a large majority of pro-inflammatory mediators remained unaltered with western diet feeding, select liver transcripts related to acute-phase inflammation signaling were altered with sub-chronic WD feeding in the setting of hepatic steatosis.

### Six weeks of WD feeding primarily affect hepatic mRNA expression patterns related to lipid metabolism and cholesterol biosynthesis

The primary finding from the current study is that sub-chronic WD feeding causes rapid alterations in select lipid and cholesterol metabolism genes which, in turn, likely contributes to the observed increases in liver fat accumulation and dysregulation of serum cholesterol levels.

The striking 28.3-fold increase in liver *Scd1* mRNA with 6 weeks of WD feeding emphasizes the potential importance of this gene in the development of NAFLD. The Scd1 enzyme catalyzes monounsaturated long-chain fatty acid synthesis from saturated fatty acyl-CoAs [21], and Scd1-deficient mice have been shown to possess less liver fat accumulation [22–24]. Indeed, pharmacologically [25] and genetically [26] knocking down *Scd1* has been shown to favorably alter fatty acid composition in mouse liver tissue. Thus, our data is in agreement with the aforementioned literature suggesting that a dietary-induced overexpression of liver *Scd1* may be crucial in the early development of NAFLD. Liver cytochrome P450 polypeptide 18 (*Cyp2c18*) was also up-regulated 2.7-fold in WD versus CTL rats. In adipocytes, *Cyp2c18* is thought to work in concert with *Scd1* to oxidize fatty acids once they are desaturated [27]. *Cyp2c18* is also thought to be involved in hepatic arachidonic acid and linoleic acid metabolism [28] and has been shown to be up-regulated in other models of NAFLD [29].

Contrary to a select number of hepatic genes being >2-fold up-regulated in WD versus CTL rats, more hepatic genes were >2-fold down-regulated in WD versus CTL rats with several of these genes being involved in cholesterol biosynthesis which is likely a partial reflection in the increased cholesterol content in the WD. Interestingly, hepatic *Tm7sf2*, which encodes the endoplasmic reticulum enzyme 3β-hydroxysterol Δ14-reductase that is involved with cholesterol biosynthesis [30], was robustly down-regulated with WD feeding. Recent evidence suggests that *Tm7sf2*^−/−^ mice present increases in NF-κB activation; this being a hallmark feature of inflammation signaling. Thus, while markers of NF-κB signaling were not altered with WD feeding in the current study, this may be an early transcriptomic event that precedes NF-κB signaling with NAFLD development. Other notable hepatic genes down-regulated in WD included: a) *Cyp51a1* and *Fdps* which have been genotype-associated with higher HDL-C levels [31] and NAFLD [32] in humans, respectively; b) *Hmgcs1* which has been shown to be positively associated with HDL-C levels [33]; and c) *Insig1* which is a gene that, when overexpressed in livers of Zucker diabetic fatty rats, attenuates hepatic steatosis [34]. Interestingly, hepatic *Pcsk9*, which encodes for a serine protease that reduces LDL receptor content [35], was paradoxically down-regulated in WD versus CTL rats. The WD-induced down-regulation in hepatic *Acly* mRNA is also paradoxical given that its inhibition in *db/db* mice has been shown to markedly protect against de novo lipogenesis and hepatic steatosis [36]. Notwithstanding, we contend that the marked dysregulation in hepatic lipid metabolism and cholesterol biosynthesis genes after 6 weeks of WD feeding is reflective of the phenotypic changes we report at the tissue level. Moreover, these genetic alterations likely precede hepatic inflammation and fibrosis development.

### Six weeks of WD feeding affect select hepatic acute phase protein and fibrosis-related mRNA expression patterns, but not local or systemic pro-inflammatory biomarkers

Various markers of liver inflammation were generally not elevated with 6 weeks of WD feeding in adult Brown Norway rats despite significant development of hepatic steatosis. These results extend mouse data to rats. Stanton et al. [16] previously demonstrated that C57BL/6 mice fed a high fat/high cholesterol diet for 6 weeks did not present increases in several pro-inflammatory liver chemokine mRNAs, but instead presented significant increases in these markers 16 and 26 weeks after feeding. Notwithstanding, we demonstrated that the mRNA expression of numerous acute phase proteins was altered (some increasing while others decreasing) following 6 weeks of WD feeding in adult rats. Acute phase proteins are proteins produced by the liver that are thought to increase or decrease in the plasma by 25 % in response to an immunological or inflammatory stimulus [37]. The presence of an acute phase response to NAFLD and NASH also extends to mouse data [38, 39]. Radonjic et al. [40] demonstrated that liver mRNAs for several acute phase reactants within days of high-fat diet feeding in ApoE3L mice. Hence, our current data suggests that liver acute phase response genes can be induced early during WD-induced NAFLD; a finding which may demarcate an early pro-inflammatory stimulus.

Interestingly, the 2-fold WD-induced increase in hepatic platelet factor 4 (*Pf4* or *Cxcl4*) mRNA may be an early indicator or even initiator of fibrosis that follows the development of NAFLD. To our knowledge, no other studies using models of NAFLD have shown this gene to change, although Pf4 has been shown to induce fibrosis [41]. Indeed, *Pf4* mRNA has been shown to be up-regulated in alcoholic hepatitis patients [42]. Recombinant murine *Cxcl4* has also been shown to stimulate the proliferation, chemotaxis, and chemokine expression of hepatic stellate cells [41]; a phenomena which suggests that there may be a potential role for its induction in the development of nonalcoholic steatohepatitis.

Of the serum pro-inflammatory cytokines measured in the current study, only serum leptin tended to be 2.4 times greater in WD versus CTL rats. Indeed longer high-fat and/or high-sugar feeding schedules have been shown to increase serum levels of select pro-inflammatory cytokines [43]. While leptin is mainly regarded as an adipokine involved with appetite regulation, some evidence supports its role in promoting an inflammation response [44, 45]. For instance, leptin-deficient *ob/ob* mice present with impairments in macrophage function [46]. Other studies have also shown that leptin is required for hepatic fibrosis development in leptin-deficient *ob/ob* mice model [47, 48]. Thus, our data supports the hypothesis that WD-induced increases in leptin levels may precede and even be permissive for subsequent liver fibrosis to occur with a longer term WD challenge.

Finally, the lack of increased NF-κB signaling in WD-fed rats may be due to the short-term feeding schedule employed in the current study. Indeed, genetically-altered murine models (*Ldlr*^−/−^, and *Apoe2*-knock-in) have been shown to present robust hepatic inflammation (via NF-κB signaling) and steatosis within 21 days of high fat/high cholesterol feeding, although wild-type mice in this same study developed only ectopic fat deposition without overt inflammation [49]. Notwithstanding, the 6-week WD feeding in the current study caused hepatic steatosis but appeared to be insufficient in duration to develop histological NASH with inflammation and fibrosis.

## Conclusions

The current study provides a unique perspective in adult rats as to how a Westernized diet affects liver transcriptomic changes over an initial 6-week feeding period. The transcriptome-wide interrogation employed herein gives insight into the rapid liver gene expression changes that occur with early WD-induced hepatic steatosis development prior to the development of secondary outcomes (i.e., inflammation and fibrosis) and support other transcriptomic work in this sub-chronic timeframe [50, 51]. This study points to the potential sensitivity of the rodent liver to dietary fat, sucrose, and cholesterol and helps add to the framework for other researchers interested in examining gene targets or metabolic pathways that occur with NASH development. Our data suggest that the expression of numerous lipid metabolism and cholesterol biosynthesis genes are rapidly altered with sub-chronic WD feeding; an effect which was manifested with significant hepatic steatosis. Furthermore, the 28-fold increase in liver *Scd1* mRNA with sub-chronic WD feeding continues to underscore the potential importance of this gene in the development of NAFLD. While WD feeding caused alterations in select acute phase response mRNAs, other pro-inflammatory signaling mediators and mRNAs appear unaltered with 6 weeks of WD feeding. Hence, this may suggest that diet-induced liver fibrosis and inflammation may either: a) occur much later with more chronic WD rat feeding paradigms; and/or b) may be initiated by other factors that precede eventual perturbations in local and systemic pro-inflammatory signalers with chronic WD feeding.

## Abbreviations

NAFLD: Nonalcoholic fatty liver disease
WD: Western diet
CTL: Control diet
Scd: Stearoyl-CoA desaturase
NASH: Nonalcoholic steatohepatitis
TG: Triglyceride
